# Artemisinin resistance without *pfkelch13* mutations in *Plasmodium falciparum* isolates from Cambodia

**DOI:** 10.1186/s12936-017-1845-5

**Published:** 2017-05-12

**Authors:** Angana Mukherjee, Selina Bopp, Pamela Magistrado, Wesley Wong, Rachel Daniels, Allison Demas, Stephen Schaffner, Chanaki Amaratunga, Pharath Lim, Mehul Dhorda, Olivo Miotto, Charles Woodrow, Elizabeth A. Ashley, Arjen M. Dondorp, Nicholas J. White, Dyann Wirth, Rick Fairhurst, Sarah K. Volkman

**Affiliations:** 1 Department of Immunology and Infectious Disease, Harvard T.H. Chan School of Public Health, 665 Huntington Avenue, I-704, Boston, MA 02115 USA; 2 Infectious Disease Initiative, The Broad Institute of MIT and Harvard, Cambridge, MA USA; 3 Laboratory of Malaria and Vector Research, National Institute of Allergy and Infectious Diseases, National Institutes of Health, Rockville, MD USA; 4 Asia Regional Centre, Worldwide Antimalarial Resistance Network, Bangkok, Thailand; 5 Nuffield Department of Medicine, Centre for Tropical Medicine and Global Health, Centre for Tropical Medicine and Global Health, University of Oxford, Oxford, UK; 6 Mahidol-Oxford Tropical Medicine Research Unit, Mahidol University, Bangkok, Thailand; 7 Centre for Genomics and Global Health, Wellcome Trust Centre for Human Genetics, University of Oxford, Oxford, UK; 8 Wellcome Trust Sanger Institute, Hinxton, UK; 9 Department of Intensive Care, Academic Medical Center, University of Amsterdam, Amsterdam, The Netherlands; 10 School of Nursing and Health Sciences, Simmons College, Boston, MA USA

**Keywords:** *Plasmodium falciparum*, Artemisinin resistance, *pfkelch13*, Piperaquine resistance

## Abstract

**Background:**

Artemisinin resistance is associated with delayed parasite clearance half-life in vivo and correlates with ring-stage survival under dihydroartemisinin in vitro. Both phenotypes are associated with mutations in the PF3D7_1343700 *pfkelch13* gene. Recent spread of artemisinin resistance and emerging piperaquine resistance in Southeast Asia show that artemisinin combination therapy, such as dihydroartemisinin–piperaquine, are losing clinical effectiveness, prompting investigation of drug resistance mechanisms and development of strategies to surmount emerging anti-malarial resistance.

**Methods:**

Sixty-eight parasites isolates with in vivo clearance data were obtained from two Tracking Resistance to Artemisinin Collaboration study sites in Cambodia, culture-adapted, and genotyped for *pfkelch13* and other mutations including *pfmdr1* copy number; and the RSA_0–3h_ survival rates and response to antimalarial drugs in vitro were measured for 36 of these isolates.

**Results:**

Among these 36 parasites one isolate demonstrated increased ring-stage survival for a *Pf*Kelch13 mutation (D584V, RSA_0–3h_ = 8%), previously associated with slow clearance but not yet tested in vitro. Several parasites exhibited increased ring-stage survival, yet lack *pfkelch13* mutations, and one isolate showed evidence for piperaquine resistance.

**Conclusions:**

This study of 68 culture-adapted *Plasmodium falciparum* clinical isolates from Cambodia with known clearance values, associated the D584V *Pf*Kelch13 mutation with increased ring-stage survival and identified parasites that lack *pfkelch13* mutations yet exhibit increased ring-stage survival. These data suggest mutations other than those found in *pfkelch13* may be involved in conferring artemisinin resistance in *P. falciparum*. Piperaquine resistance was also detected among the same Cambodian samples, consistent with reports of emerging piperaquine resistance in the field. These culture-adapted parasites permit further investigation of mechanisms of both artemisinin and piperaquine resistance and development of strategies to prevent or overcome anti-malarial resistance.

**Electronic supplementary material:**

The online version of this article (doi:10.1186/s12936-017-1845-5) contains supplementary material, which is available to authorized users.

## Background

Artemisinin (ART) and related compounds provide the main class of anti-malarial drugs, and ART resistance in *Plasmodium falciparum* is one of the greatest threats to global efforts to control, eliminate and eradicate malaria. To forestall emergence and spread of ART resistance it was recommended that ART and its derivatives be used only in combination with a partner drug as an ART combination therapy (ACT), with over 400 million ACT treatments dispensed annually. It is conservatively estimated that 116,000 additional deaths would occur annually in the event of widespread ART resistance, with annual health costs of US$32 million and productivity losses exceeding US$385 million [1]. The magnitude of the health and economic threat posed by ART resistance serves as an urgent call to action to develop strategies that circumvent its spread [2]. Doing so requires understanding the underlying mechanisms of ART resistance.

Currently, ART resistance is prevalent across the Greater Mekong Sub-region (GMS) and centered on Cambodia, where it was first detected in 2007 [3, 4]. Concern for the spread of ART resistance outside of Southeast Asia led to the Tracking Resistance to Artemisinin Collaboration (TRAC) study that assessed and tracked ART resistance across 15 sites both in Asia and Africa [5]. Using parasite clearance as a measure of resistance, the TRAC project confirmed that ART-resistant *P. falciparum* was established in Cambodia, Laos, Myanmar, Thailand, and Vietnam.

Artemisinin resistance is indicated by either delays in parasite clearance from patients, or by increased in vitro parasite survival under dihydroartemisinin (DHA) in ring-stage survival assay (RSA_0–3h_) [6]. Sequencing parasites selected under increasing ART pressure identified *pfkelch13* as a critical gene for conferring ART resistance [7]. Multiple mutations have been identified in this gene, and several key single nucleotide polymorphisms (SNPs) have been validated using a gene-editing approach [8, 9]. However, the function of *Pf*Kelch13 and its role in ART resistance remain unclear.

Recent reports confirm that specific *pfkelch13* mutations confer ring-stage survival [7, 8], and indicate possible mechanisms of ART resistance, including perturbation of haemoglobin processing [10–12], protein ubiquitination [13], increased expression of oxidative stress response [13, 14], unfolded protein response pathways [14], or phosphatidylinositide 3-kinase pathways [15]. While these results may all be relevant, a full understanding of the mechanisms involved in ART action and resistance has yet to emerge [16, 17].

One of the key partner drugs used with ART for malaria treatment in the GMS is piperaquine (PPQ), which has proved well tolerated and highly effective in areas where multi-drug-resistant *P. falciparum* is prevalent. However, emergence of PPQ resistance threatens to undermine this strategy in areas of increasing ART resistance [18, 19]. Currently PPQ resistance is evident by higher PPQ half maximal effective concentration (EC_50_) values and elevated recrudescence rates in settings where DHA–PPQ is in use and ART resistance is common [18, 19]. Reported molecular markers of PPQ resistance include SNPs in *pfcrt* and de-amplification of a region on chromosome 5 that includes or is proximal to *pfmdr1* [20]. PPQ resistance is also associated with an amplification of *plasmepsin II* and *III* [21, 22]. Yet, to date no mechanism has been established for PPQ resistance. Increasing resistance to both ART and PPQ in the same parasite population has motivated a call for use of triple combination therapy in certain malaria-endemic settings where ACT may have reduced efficacy [18, 23]. Specifically, mefloquine (MFQ) has been suggested for combination with ART and PPQ given the inverse effects PPQ and MFQ pressure have on *pfmdr1* copy number variation (CNV), as PPQ negatively selects *pfmdr1* CNV, while MFQ positively selects *pfmdr1* CNV [18].

As part of the TRAC collaboration, 157 cryopreserved parasites were obtained from two sites in western Cambodia with a range of in vivo clearance phenotypes. Of these parasites, 68 were culture-adapted, and a sub-set of 36 parasites evaluated for their RSA_0–3h_ phenotype. Using a high-resolution melt (HRM) genotyping assay for the most common *pfkelch13* mutation (C580Y) and Sanger sequencing, *pfkelch13* propeller mutations were tested for associations with both parasite in vivo clearance half-lives and in vitro parasite RSA_0–3h_ survival rates. The analysis (1) identified parasites lacking *pfkelch13* mutations, but exhibiting increased RSA_0–3h_ survival phenotype (referred to as discordant parasites), suggesting that loci other than *pfkelch13* may be involved in ART resistance; (2) demonstrated a new association between D584V and increased ring-stage survival; and, (3) detected a PPQ-resistant isolate among these parasites, consistent with other reports from this malaria-endemic region. Although there is a strong association between *pfkelch13* mutations and increased ring-stage survival, mutations in *pfkelch13* are not necessary for this ART resistance phenotype. Furthermore, in vitro PPQ resistance is present among these culture-adapted Cambodian parasites, which will enable further investigation of PPQ resistance mechanisms.

## Methods

These parasite samples were obtained with informed consent from patients enrolled in the TRAC study in the Pailin and Pursat sites located in Western Cambodia. Full details of this study, the approvals, and the clinical and laboratory methodologies have been reported in detail elsewhere [5].

### Culture-adaptation and maintenance of TRAC parasites

All parasite samples were collected under protocols approved by ethical review boards in Cambodia, at Oxford University and at the Harvard T. H. Chan School of Public Health. Culture-adaptation of parasites was accomplished by thawing cryopreserved material containing infected red blood cells (iRBCs) that had been mixed with glycerolyte. Parasites were maintained in fresh human blood (O+) and Hepes buffered RPMI media containing 12.5% AB+ human serum (heat inactivated and pooled). Cultures were placed in modular incubators and gassed with 1% O_2_/5% CO_2_/balance N_2_ gas and incubated with rotation (50 rpm) in a 37 °C incubator (Additional file 1).

### Sample extraction

Genetic material were extracted from filter papers (Whatman) and culture-adapted material using Promega DNA IQ Casework Pro Kit for Maxwell 16 (Promega Corp., Madison, WI, USA) and Qiagen (QIAmp DNA Blood Mini Kit) commercial kits, respectively, according to manufacturer instructions.

### Genotyping

Development of a high-resolution melt (HRM) assay to screen populations for mutations around amino acid position 580 in the *pfkelch13* locus. The forward primer (5′-GGCACCTTTGAATACCC-3′), reverse primer (5′-CATTAGTTCCACCAATGACA-3′), and unlabeled, blocked probe (5′-AGCTATGTGTATTGCTTTTGAT-block-3′) were amplified asymmetrically at 0.5, 0.1, and 0.4 μM, respectively with 1 ng template DNA. After an initial 2-min hold at 95 °C, 5 or 10 μL reaction mixtures with 2.5 × HRM master mix (BioFire Defense, Salt Lake City, UT. USA) were PCR amplified for 55 cycles: 95 °C for 30 s, 66 °C for 30 s, and 74 °C for 30 s, followed by a pre-melt step of 95 and 28 °C for 30 s each. Products were melted from 45 to 90 °C on a BioFire Defense LightScanner-384 or -32 and analysed using the manufacturer software. Two plasmid controls containing the wild-type and mutant alleles were included as standards for every HRM run. Molecular barcoding was performed as described [24, 25] to identify monogenomic samples with unique parasite genotypes.

### Whole genome sequences

This publication uses sequencing data generated by the Pf3k project [26]. The variant call files generated from this project were used to identify SNPs in each of the samples used in this study.

### *Pfkelch13* PCR sequencing strategy

The propeller domain of *pfkelch13* was PCR amplified using Phusion HF DNA Polymerase kit and primers 3F and 1R′ in all 68 culture adapted parasites. DNA from KH001_024 was also amplified with primers 4F and 3R′. An aliquot of PCR product was resolved by gel electrophoresis to check for specificity and yield and the remaining product was purified using DNA Clean & Concentrator, ZymoResearch and sequenced using the same primers used to amplify the product by Genewiz. The full ORF of *pfkelch13* was PCR amplified using primers 1F and 1R from 3D7 and individual culture-adapted parasites. The resulting PCR product of ~2.2 kb was purified by gel extraction (QIAquick Gel Extraction Kit, Qiagen) and sequenced at Genewiz using primers 1F, 2F, 3F, 1R, 2R and 3R. Primer sequences are as follows: 1F: 5′-ATGGAAGGAGAAAAAGTAAAAACAAAAGCAAATAG-3′; 2F: 5′-GGTAGGTGATTTAAGAATTACATTTATTAATTGGT-3′; 3F: 5′-CATTCCCATTAGTATTTTGTATAGGTG-3′; 4F: 5′-GTAGAGGTGGCACCTTTGAATACCCCTAGATCATC-3′ 1R: 5′-TTATATATTTGCTATTAAAACGGAGTGACCAAATCTG-3′; 1R′: 5′-TTA TAT ATT TGC TAT TAA AAC GGA GTG-3′; 2R: 5′-AGCCTTATAATCATAGTTATTACCACCAAAAACG-3′; 3R: 5′-TGTTGGTATTCATAATTGATGGAGAATTC-3′; 3R′: 5′-ATAAAATGTGCATGAAAATAAATATTAAAG-3′.

### In vitro 72-h drug susceptibility by SYBR green staining

Drug susceptibility was measured using the SYBR Green I method as previously described [27]. Briefly, tightly synchronized 0–6 h rings were grown for 72 h in the presence of different concentrations of drugs in 384-well plates at 1% haematocrit and 1% starting parasitaemia; and, growth at 72 h was measured by SYBR Green staining of parasite DNA. Except for PPQ and KH001_053 where a 24-point dilution series was used, a 12-point dilution series of each drug was carried out in triplicate and repeated with at least three biological replicates. DMSO stocks of drugs were dispensed by a HP D300 Digital Dispenser (Hewlett Packard Palo Alto, CA, USA) except for the CQ and PPQ stocks that were prepared in water and dispensed with a Velocity 11 Robot (Bravo). Relative fluorescence units (RFU) was measured at an excitation of 494 nm and emission of 530 nm on a SpectraMax M5 (Molecular Devices Sunnyvale, CA, USA) and analysed using GraphPad Prism version 5 (GraphPad Software La Jolla, CA. USA). EC_50_ values were determined with the curve-fitting algorithm log(inhibitor) vs response—variable slope, except for PPQ and KH001_053. Due to the bimodal dose response of KH001_053 to PPQ, curve fitting didn’t give an accurate EC_50_ value. The reported PPQ EC_50_s for KH001_053 are estimates using biphasic curve fitting. Spearman correlation analysis was performed to assess the relationship between the anti-malarial EC_50_ values and in vivo clearance half-life, ring survival assay value or *pfmdr1* copy number. p values <0.05 were considered significant.

### Copy number variation assays

To determine copy numbers for *pfmdr1, plasmepsin II* and the 63 kb amplicon genes (PF3D7_0520100, PF3D7_0520500, PF3D7_0520600, PF3D7_0520900 and PF3D7_0521000), real time quantitative PCR was performed on genomic DNA (extracted with QIAmp Blood Mini Kit, Qiagen) as previously described [28] with the following modifications: Amplification reactions were done in MicroAmp 384-well plates in 10 μL SYBR Green master mix (Applied Biosystems), 150 nM of each forward and reverse primer and 0.4 ng template. Forty cycles were performed in the Applied Biosystems ViiA™ 7 Real-time PCR system (Life Technologies). Forward and reverse primers used were as previously described to amplify the following loci: *pfmdr1* (PF3D7_0523000) [29], the 63 kb region on chromosome 5 (PF3D7_0520100, PF3D7_0520500, PF3D7_0520600, PF3D7_0520900 and PF3D7_0521000 [20]) and *plasmepsin II* (PF3D7_140800) [22]. For the endogenous controls, *β*-*tubulin* forward and reverse primers [28] were used for *pfmdr1,* PF3D7_0520100 and PF3D7_0520900 while *pfldh* forward and reverse primers [30] were used for PF3D7_0520500, PF3D7_0520600 and PF3D7_0521000. Target primers used were validated to have the same PCR efficiencies as their endogenous control primers; and, average copy number values were calculated for each gene using data from three independent experiments.

### Sequencing the *pfcrt* locus

The entire *pfcrt* locus was sequenced as previously described [20] with some modifications. Briefly, total RNA was extracted using RNeasy kit (Qiagen) and used to generate cDNA using Superscript III (Invitrogen). The resulting cDNA was then used as template for PCR amplification of *pfcrt* [20], followed by Sanger sequencing (GENEWIZ) [20]. Sequence data analysis was performed using MacVector.

### Ring survival assay (RSA_0–3h_)

The RSA_0–3h_ was performed as described previously [6]. Essentially, parasites were sorbitol synchronized twice at 40-h intervals, synchronous 40–44 h segmented schizonts were incubated for 15 min at 37 °C in serum-free media supplemented with heparin to disrupt agglutinated erythrocytes and late stages were purified with 35/65% discontinuous Percoll gradient. The segmented schizonts were washed and cultured with fresh RBCs for 3 h, after which late stages were removed by sorbitol treatment. Cultures with 0–3 h rings were adjusted to 2% haematocrit and 1% parasitaemia and seeded into a 24-well plate with 1 ml complete media per well. To these wells, either DHA at 700 nM or 0.1% DMSO were added immediately and incubated for 6 h at 37 °C, washed and incubated in drug free media. At 72 h from seeding, thin blood smears were made from control and treated wells and survival rates were measured microscopically by counting the proportion of next generation viable rings with normal morphology. Survival rates were expressed as ratios of viable parasitaemias in DHA-exposed and DMSO-treated controls. Parasites were counted from 10,000 RBC, and two separate individuals served as independent slide readers.

## Results

### Characteristics of original and culture-adapted parasite isolates

A total of 157 cryopreserved TRAC study samples collected in 2011 from Pursat or Pailin, Cambodia where ART resistance is observed were obtained. These isolates were selected before the discovery of the *pfkelch13* marker and were designed to comprise western Cambodian samples, with two-thirds of the samples coming from the upper segment of the clearance half-life distribution and one-third being isolates with the shortest clearance times. 68 parasites were culture-adapted for further investigation, without knowledge of their in vivo clearance data. A summary of the overall population and the sub-set selected for culture-adaptation and further characterization is provided in Additional files 2 and 3. Collectively, 63% of both the original and the adapted parasites were from Pursat and the remaining 37% were from Pailin (Additional file 3). Similarly, 67% of both parasite sets (original and adapted) had a delayed clearance of ≥5 h and 33% had a clearance half-life of <5 h; for one isolate there were insufficient data to determine an in vivo half-life. All culture-adapted samples were confirmed to harbour monogenomic infections by molecular barcode analysis [25], only those parasites with unique barcode signatures [25] were initially pursued to maximize genetic diversity and exclude highly similar parasites among the analysed isolates.

### *Pfkelch13* propeller mutations are associated with parasite clearance half-life ≥5 h

Next, parasites were genotyped for the *pfkelch13* locus previously associated with delayed parasite clearance (defined as >5 h) and increased ring-stage survival (defined as RSA_0–3h_ ≥1%) [7]. An HRM genotyping assay [24, 25] for the common non-synonymous mutation conferring the C580Y change in *Pf*Kelch13 was developed and validated, among the 157 original samples with an unambiguous genotyping call, 71% (108/152) had the C580Y mutation (Additional file 2). These data, confirmed a significant positive association between the C580Y non-synonymous mutation and in vivo parasite clearance half-life values for these parasites [26]. This association was more pronounced among Pursat parasites (unpaired Student’s t test with Welch’s correction, p < 0.0001) as compared to parasites from Pailin (p = 0.0322, Additional file 4) for this sample set.

**Fig. 1:**
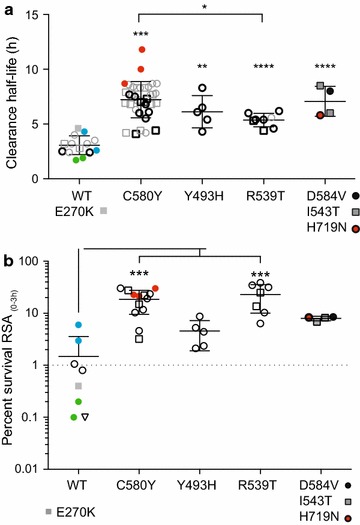
Positive association between *pfkelch13* propeller mutations and in vivo clearance half-life as well as in vitro RSA_0–3h._ Parasites from Pursat (*round symbols*) or Pailin (*square symbols*) were classified according to their *pfkelch13* alleles. **a** Comparison of clearance half-live values (hours, h) from culture-adapted parasites harbouring different *pfkelch13* propeller alleles, and combining alleles represented by less than five samples. Parasites outlined in bold were further tested for RSA_0–3h_ phenotypes in **b**. A one-way ANOVA test, with a Tukey’s post-test, was performed between wild-type (WT) and each allele category. Significance values are indicated by *asterisks*: *(p < 0.05); **(p < 0.01); ***(p < 0.001); and ****(p < 0.0001). **b** In vitro RSA_0–3h_ testing. Percent survival is displayed on the y-axis and *pfkelch13* allele represented on the x-axis. All parasites that harbour a *pfkelch13* propeller mutation exhibit an RSA_0–3h_ value of >1% (*dotted line*). Data were analysed by Kruskal–Wallis test followed by Dunn’s multi-comparison test. p values: ***<0.001. An additional subset represented by *coloured symbols*, also underwent conventional in vitro drug testing and assessment of *pfmdr1* copy number variation. These were chosen as representatives of parasites that were ART sensitive (*green*) or, ART resistant (*red*) as assessed by both RSA_0–3h_ phenotype and *pfkelch13*. Two ‘discordant’ samples (resistant according to RSA but wild-type at *pfkelch13*) are indicated in *blue*

A combination of available whole genome sequence (WGS) data from the original samples [26, 31, 32], and *pfkelch13*-specific PCR-based sequencing identified both *pfkelch13* mutations other than C580Y in this population and confirmed persistence of all *pfkelch13* mutations in the culture adapted parasites further analysed. Overall there was good concordance between the molecular barcode derived from filter paper and cultured parasite samples, and between the *pfkelch13* mutations identified in WGS or PCR re-sequencing data. Six different mutations were present in the *pfkelch13* propeller domain: Y493H, R539T, I543T, C580Y, D584V, and H719N, and two *pfkelch13* mutations outside the propeller region: H136N, E270K. The two parasites with the H136N change also contained C580Y, but E270K was seen by itself, in a single isolate (Additional file 2). All propeller domain mutations were positively associated with in vivo clearance values (Fig. 1a), including parasites with the double mutation H136N/C580Y genotype (Additional file 2). Additionally, two asparagine (Asn) insertions at codon 142 were identified in all isolates tested except those with the Y493H change and one parasite with the D584V genotype (Additional file 2). The location of this Asn insertion was distinct from that previously noted among Asian parasites [33]. No association between this Asn insertion and either parasite clearance half-life or RSA_0–3h_ phenotype was detected.

The sub-set of 68 culture-adapted parasites harbored all mutation types observed in the 157 initial isolates except H136N, and each *pfkelch13* mutation detected by WGS was confirmed by PCR re-sequencing (Additional files 2, 3). Overall, the sub-set of culture-adapted parasites showed similar distribution of *pfkelch13* genotypes detected among the original 157 isolates chosen for this study. The majority of parasites in this sub-set (79% or 54/68) carried non-synonymous mutations in *pfkelch13,* with the most common change resulting in C580Y (53% or 36/68, Table 1). Among these culture-adapted parasites from Pailin there was a higher frequency of *pfkelch13* mutations than parasites from Pursat (88 and 74%, respectively), with the C580Y mutation being the most prevalent (77 and 60%, respectively). Both geographic locations had their own private mutations not found in the other location (Additional file 2). All *pfkelch13* propeller mutations displayed positive associations with in vivo clearance phenotypes (Fig. 1a).

**Table 1:** Summary of *Pf*Kelch13 mutations

Culture adapted parasites	Total	WT	Y493H	R539T	I543T	C580Y	D584V	H719N	H136N/C580Y	E270K	Asn Insert
Pailin	25	3	0	2	2	17	0	0	0	1	6
Pursat	43	11	5	6	0	19	1	1	0	0	19
Total	68	14	5	8	2	36	1	1	0	1	25

### *Pfkelch13* propeller mutations are positively associated with increased ring-stage survival by in vitro RSA_0–3h_

There was a positive association between increased RSA_0–3h_ survival phenotypes and *pfkelch13* mutations among 36 culture adapted parasites (Fig. 1b) and parasites with the D584V change previously associated with increased in vivo parasite clearance time [5] demonstrated an increased ring-stage survival by RSA_0–3h_.

In vitro RSA_0–3h_ phenotypes were evaluated as a measure of ART resistance for 36 adapted parasites exhibiting a range of in vivo clearance phenotypes and harbouring a variety of *pfkelch13* mutations. Consistent with previous reports [6], the distribution of RSA_0–3h_ phenotypes spanned two log_10_ ranges for all parasites analysed. Overall, there was a significant difference between wild-type (RSA_0–3h_ = 0.1–6.0%, n = 7) parasites and those with *pfkelch13* mutations (RSA_0–3h_ = 2.1–38.6%, n = 29) in terms of RSA_0–3h_ parasite survival rate (Student’s t test p = 0.0018). RSA_0–3h_ survival rates also differed between parasites with individual *pfkelch13* mutations; whereby parasites with either a C580Y or R539T mutation exhibited the highest survival rates (Fig. 1b). One isolate with a *Pf*Kelch13 E270K mutation outside the propeller domain exhibited a clearance of >4 h, yet had an RSA_0–3h_ value of 0.4%. Among the parasites with RSA_0–3h_ values >1%, 19% (7/36) had in vivo clearance values between 4 and 5 h, which suggests that for this specific population a clearance value of 4 h or greater is more consistent with ART resistance based upon an RSA_0–3h_ phenotype (Additional file 5).

All *pfkelch13* propeller polymorphisms found among these parasites were previously identified [7, 33]. However, a novel positive association was demonstrated between the D584V mutation and in vitro ring survival values (8.0 h clearance and 8.5% RSA survival for D584V), not previously tested for in vitro ART resistance (Fig. 1b). These data confirm that all *pfkelch13* propeller mutations found in these TRAC isolates are associated with an RSA_0–3h_ phenotype ≥1%, thus considered in vitro ART resistant.

### Identification of discordant parasites that exhibit increased ring-stage survival but lack *Pfkelch13* mutations

ART resistance in vitro has been suggested to correlate with an RSA_0–3h_ survival value of ≥1% [6], and all isolates in this study with a mutation in *pfkelch13* had an RSA_0–3h_ ≥1%. All wild-type *pfkelch13* parasites had in vivo clearance half-lives below 5 h (Additional file 2); however, there was a range of in vitro RSA_0–3h_ survival phenotypes from 0.1 to 6%, including four isolates with RSA_0–3h_ of approximately ≥1% (0.8, 1.1, 3, and 6%) (Fig. 1b). Parasites with an RSA_0–3h_ of ≥0.8% that lack *pfkelch13* mutations are considered discordant. These data suggest changes outside *pfkelch13* could also confer an increased ring-stage survival phenotype indicative of ART resistance in this parasite population.

Using WGS data previously generated [26] for 33 of the RSA_0–3h_ phenotyped parasites, the question was explored whether mutations outside *pfkelch13* were evident in the four parasites with RSA_0–3h_ ≥0.8% values that lack *pfkelch13* ORF mutations. This small data set was underpowered to identify novel mutations using a genome-wide strategy, but a candidate gene approach was undertaken to ask whether any known drug resistant loci or other previously identified secondary mutations could potentially explain the high RSA_0–3h_ survival values (Additional file 6). Variant positions were identified across 23 genes previously associated with ART resistance among the set of 33 RSA_0–3h_ phenotyped parasites [7, 15, 26], which included both sensitive and resistant parasites. Examination of both sensitive and resistant parasites would allow detection of mutations that may account for the differences in RSA_0–3h_ survival values and identification of mutations that confer high RSA_0–3h_ survival values independent of *pfkelch13*.

This analysis identified 37 SNPs (30 non-synonymous, four synonymous, two intronic) spread across 19 unique genes (Additional file 7). Eleven of these positions were invariant among these four isolates that lack *pfkelch13* ORF mutations but exhibited an RSA_0–3h_ survival value ≥0.8%. These 37 SNP positions are found in genes known to be associated with drug resistance including dihydrofolate reductase (*dhfr*, PF3D7_0417200) [34] and dihydropteroate synthase (*dhps*, PF3D7_0810800) [35]. Another locus, phosphatidylinositol-4-phosphate 5-kinase (PIP5K, PF3D7_0110600) [36], is involved in cellular signaling pathways, including synthesis of PIP2, a substrate for PI3K to produce PIP3, which activates the AKT family of serine/threonine kinases, a pathway implicated in an ART resistance mechanism [15]. The putative NLI Interacting factor-like phosphatase (NIF4, PF3D7_1012700) [36–39] is a protein phosphatase 2C protein. Several members of the *Plasmodium* phosphatome contain *kelch* domains and have been shown to be key regulators of parasite development and differentiation [37, 40], but NIF4 lacks an intact DxDx(T/V) motif, and thus may be catalytically inactive [39]. *Pfmdr2* (PF3D7_1447900) [41, 42] is implicated as a secondary locus associated with delayed parasite clearance [26]. No other changes associated with ART resistance reported in the literature [43, 44] were detected among these parasites (Additional file 6).

### Detection of PPQ resistance phenotype

**Fig. 2:**
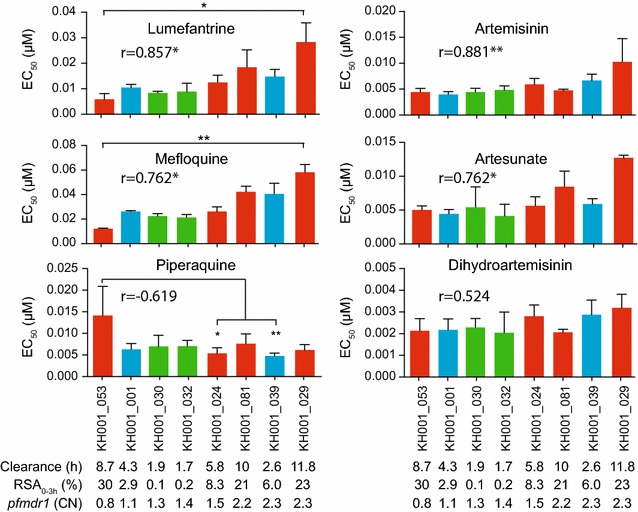
*Pfmdr1* copy numbers are positively associated with MEF, LUM, ART and ARS. Drug sensitivity assays were set up with 0–6 h old ring stage parasites and % growth was measured 72 h later by SYBR *Green*. Shown are the average EC_50_ values from at least three biological replicates for two ART sensitive (*green*), two discordant (*blue*) and four resistant (*red*) isolates. Their respective clearance time, RSA_0–3h_ percent survival and their *pfmdr1* copy number are indicated *at the bottom*. The Spearman correlation coefficient for *pfmdr1* copy number and each drug is shown in the graph. A one-way ANOVA test, with a Tukey’s post-test was performed between all isolates for each drug. Significance values are indicated by *asterisks*, as follows: *(p < 0.05); **(p < 0.01)

To further evaluate the relationship between RSA_0–3h_ phenotype and *pfkelch13* mutations, a sub-set of eight isolates with a range of ring-stage survival phenotypes was selected. This sub-set included the two most sensitive (in green) and the two most resistant (discordant, in blue) parasites without *pfkelch13* mutations, as well as four resistant parasites that contain *pfkelch13* mutations (in red) (Fig. 1b). First standard in vitro drug tests [27] were used to evaluate anti-malarial drug responses among these parasites (Fig. 2). EC_50_ values for ART and its derivatives did not consistently correlate with either the in vivo clearance or in vitro RSA_0–3h_ phenotypes, as previously reported [6, 7] (Additional file 8; Fig. 2). The parasite clearance half-life and RSA_0–3h_ survival phenotypes did not consistently associate with responses to other anti-malarial compounds tested (chloroquine (CQ), MFQ, lumefantrine (LUM), atovaquone (ATV), quinine (QN) and PPQ, Additional file 8). A significant positive correlation was observed between EC_50_ values for ART (r = 0.881, p = 0.0072), artesunate (AS, r = 0.719, p = 0.0368), MFQ (r = 0.7619, p = 0.0368) and LUM (r = 0.857, p = 0.0107) and increased *pfmdr1* copy number (Spearman correlation, Additional file 8 and Fig. 2), consistent with previous reports [30, 45, 46].

**Fig. 3:**
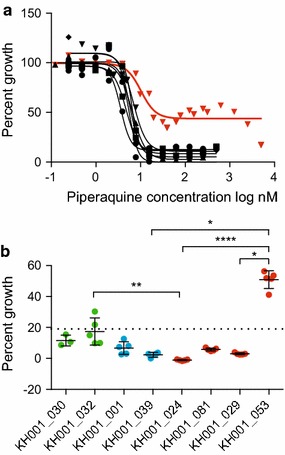
KH001_053 exhibits resistance to piperaquine (PPQ). **a** EC_50_ growth curves for eight parasite isolates in different PPQ concentrations. Percent growth is displayed on the y-axis and the PPQ concentration (nM) is represented in a log scale on the x-axis, with KH001_053 indicated by *red triangles*. **b** Relative growth is shown (percent growth) for TRAC isolates exposed to 125 nM PPQ for 72 h starting with 0–6 h old rings, with individual parasite lines represented on the x-axis. *Colours* correspond to parasites with RSA_0–3h_ ≤0.2% that lack *pfkelch13* mutations (sensitive, *green*); RSA_0–3h_ >3% that lack *pfkelch13* mutations (discordant, *blue*); and, RSA_0–3h_ >1% that harbour with *pfkelch13* mutations (resistant, *red*). Data were analysed by Kruskal–Wallis test followed by Dunn’s multi-comparison test. p values: *<0.1, **<0.01, ****<0.0001

Interestingly, one isolate, KH001_053, with a slow clearance time (8.7 h) and high ring survival (30%) exhibited a bimodal response to PPQ that indicated parasite survival at high PPQ concentrations (Fig. 3a). Compared to other isolates, KH001_053 also demonstrated a high survival phenotype (50–70%) when grown at high concentrations of PPQ (125 and 250 nM) for 72 h [47] (Fig. 3b). This PPQ-resistant parasite also demonstrated hyper-susceptibility to MFQ and LUM (Fig. 2), explicable at least in part by a single copy of the *pfmdr1* gene (Additional file 8).

KH001_053 was then tested for evidence of known molecular markers associated with PPQ resistance [20], including C101F mutation in *pfcrt*, an amplification of 63 kb region on chromosome 5, and amplification of *plasmepsin II* [21, 22]. All eight isolates were evaluated for SNPs in the *pfcrt* locus by PCR amplification and sequencing of cDNA across *pfcrt*. Eight non-synonymous mutations (M74I, N75E, K76T, A220S, Q271E, N326S, I356T, R371I) were found that were fixed in these eight isolates. No evidence of the previously reported C101F haplotype in the KH001_053 parasite was detected [20]. Since PPQ resistance has been associated with an increased copy number of a 63 kb amplicon on chromosome 5 (including PF3D7_0520100, PF3D7_0520500, PF3D7_0520600, PF3D7_0520900 and PF3D7_0521000, 825600–888300 bp) and a decreased number of *pfmdr1* in vitro [20, 30] as well as an increased copy number of *plasmepsin II* (PF3D7_140800) locus in vivo [21, 22], copy number assays were performed on those loci. In concordance with to a previous study [30] no increased copy number on the 63 kb amplicon region on chromosome 5 was detected. However, KH001_053 did harbour two copies of *plasmepsin II* consistent with previous reports where the presence of an increased *plasmepsin II* and *III* copy number was predictive of recrudescence in DHA/PPQ-treated patients [21, 22]. These findings strengthen the hypothesis that increased *plasmepsin II* copy number may play a role in PPQ resistance.

## Discussion

Within a set of 68 culture-adapted Cambodian parasites, some parasites exhibited an increased RSA_0–3h_ phenotype that lack *pfkelch13* mutations, a parasite harbouring the *Pf*Kelch13 D584V change exhibited increased RSA_0–3h_ survival, and some parasites showed evidence for PPQ resistance. To evaluate a set of 157 Cambodian *P. falciparum* isolates from the TRAC study, with known clearance half-life data, a genotyping assay for the common C580Y change associated with ART resistance was developed and confirmed a clear association between delayed parasite clearance half-life and the presence of the C580Y allele. Sequence or genotype data from 146 of these parasites demonstrated a positive association between the clearance phenotype and *pfkelch13* propeller mutations. 68 of these parasites were culture-adapted and 36 were tested for their in vitro RSA_0–3h_ phenotype. Increased parasite clearance half-life was associated with RSA_0–3h_ ≥1% values, with a 4-h or greater clearance phenotype being more consistent with ART resistance based upon RSA_0–3h_ for this population. A novel positive association was demonstrated between D584V and in vitro ring-stage parasite survival under DHA among these parasites.

Despite this general correspondence between in vivo clearance and in vitro RSA_0–3h_ phenotypes and *Pfkelch13* propeller mutations, there were discordant parasites that exhibited a resistant RSA_0–3h_ phenotype (RSA_0–3h_ ≥0.8%) yet harboured no mutations in the entire *pfkelch13* ORF, consistent with other studies that found similarly discordant parasites by RSA_0–3h_ [48], or by clearance half-life [26]. A parasite (see Additional file 5) with a *Pf*Kelch13 E270K outside the propeller domain exhibited a clearance value of >4 h, yet had an RSA_0–3h_ value of 0.4 %. These differences may be due to differential host factors including variances in drug pharmacokinetics, pharmacodynamics or host immune status. More isolates with an E270K mutation need to be studied to confirm the role of a *pfkelch13* mutation outside the propeller domain in ART resistance and reduce the probability of an artifact related to microscopy. Taken together, these results suggest that ART resistance in natural parasite populations could be mediated by changes outside of the *pkelch13* propeller domain. Parasites with these phenotypes around the defined cut off values may be important for identification and study of loci other than *pfkelch13* that contribute to ART resistance. Inspection of candidate loci, including traditional drug resistance mutations, recently identified secondary mutations, or loci related to pathways implicated in ART resistance, identified a few specific changes that may account for these discordant parasites, but lacked the statistical power to determine whether any of these contributed to ART resistance. However, identification of potential mutations in the *PIP5K* and *pfmdr2* loci is consistent with other reports. Nevertheless, the identification of parasites that lack *pfkelch13* mutations yet harbour an increased RSA_0–3h_ survival phenotype, suggest loci other than *pfkelch13* may modulate ART resistance in these parasites. Alternative strategies, such as the use of independent chemogenomic strategies or genetic crosses in vitro [49], might be useful to identify loci involved in conferring the observed increased ring-stage survival phenotype in the absence of *pfkelch13* mutations.

No relationship was found between EC_50_ values for ART or its derivatives with either clearance or ring-stage survival phenotypes, but a previously noted correspondence was confirmed between *pfmdr1* copy number and EC_50_ values of ART and AS, as well as with MFQ and LUM responses, among eight parasites evaluated for drug responses (Fig. 2). Drug testing identified a PPQ-resistant isolate among this population, consistent with reports in the literature of increased PPQ resistance in this region [18, 19]. The PPQ-resistant parasite had a single copy of the *pfmdr1* gene [20, 30] and amplification of the *plasmepsin II* locus previously noted [21, 22]. The exact nature of this resistance, the role of *plasmepsin II* and the explanation for the apparent bimodal response to PPQ remains unknown and under investigation, but identification of culture-adapted parasites affords additional testing of both the phenotype and genotype (Additional file 1).

It has been suggested [18, 19] that PPQ resistance arose in the context of or as a consequence of ART resistance. Understanding the nature of this resistance and the underlying mechanism will be critical for reducing or restricting emergence of PPQ resistance in Southeast Asia where reports indicate rapid emergence over the past several years [18]. Furthermore, since DHA–PPQ is being utilized in mass drug administration campaigns designed to facilitate elimination of malaria in specific settings, use of molecular markers of resistance and understanding the relationship between ART and PPQ resistance will be important. Thus, identification of PPQ resistance with MFQ and LUM hyper-susceptibility in an ART resistant parasite could be an important clue to understanding mechanisms of drug response, and testing the implications of triple therapy, such as ART–PPQ–MFQ, being considered for use in this region [18].

## Conclusions

In a large set of *P. falciparum* isolates from the TRAC study, the associations between C580Y and several other *pfkelch13* propeller mutations and parasite clearance half-life was investigated. In a subset of 68 culture-adapted parasites, RSA_0–3h_ survival and conventional responses to multiple antimalarial drugs were measured. Several *pfkelch13* mutations (including D584V) were associated with increased RSA_0–3h_ survival, and discordant parasites with RSA_0–3h_ survival 1% but without *pfkelch13* ORF mutations were identified. These data suggest that mutations outside of *Pfkelch13* may confer in vitro ART resistance in *P. falciparum*. It will therefore be important to continue phenotypic assessment of ART resistance, in addition to surveying for *pfkelch13* propeller mutations. Detection of a PPQ-resistant parasite will enable further studies to investigate underlying mechanisms of PPQ resistance. This panel of culture-adapted parasites with known parasite clearance half-life, RSA_0–3h_ survival, and *pfkelch13* genotype will facilitate further investigation of ART resistance mechanisms, providing tools to identify potential *Pf*Kelch13-binding partners and other interacting molecules.

## Additional files

**Additional file 1.** Culture-adaptation of TRAC parasites.

**Additional file 2.** Summary of all genotypes available.

**Additional file 3.** Schematic shows distribution of parasites obtained from Pursat (blue star on map, KH1 parasites indicated in blue) and Pailin (red star, KH4 parasites indicated in red) from Cambodia (map modified from https://commons.wikimedia.org/wiki/File:Cambodia_provinces_en.svg).

**Additional file 4.** Distribution of C580Y mutations in Cambodian isolates. Parasites from Pursat (round symbols) or Pailin (square symbols) were classified according to their *Pfkelch13* alleles. Comparison of clearance half-life values (hours, h) from parasites harboring the wild-type (C) or mutant (Y) allele at amino acid position 580 in the Pfkelch13 locus, with culture-adapted parasites represented by black outlined symbols. C580 parasites harboring a mutation in the Pfkelch13 propeller domain other than C580Y are indicated by grey filled symbols. An unpaired t test was performed between the C580 and C580Y carrying parasites from Pursat (p < 0.0001) or Pailin (p < 0.05); with significant differences (p < 0.0001 overall) when both Pursat and Pailin were combined.

**Additional file 5.** Association between percent survival RSA_0–3h_ and clearance time. Correlation between clearance time on the x-axis and percent survival RSA_0–3h_ on the y-axis. Each dot represents an isolate. Samples represented by color were used for in vitro drug testing and for *pfmdr1* copy number variation determination—green for ART sensitive; blue for discordant; and red for ART resistant parasites. The grey dot marks the isolate with an E270K mutation. The dotted lines represent the cut off used to discriminate between resistant and sensitive parasites (1% for RSA_0–3h_, 4 or 5% for clearance).

**Additional file 6.** All genes analysed for candidate gene approach.

**Additional file 7.** All positions that are variant in RSA_0–3h_ phenotyped isolates.

**Additional file 8.** Spearman correlation coefficients between antimalarial EC50 values, in vivo clearance half-life, ring survival assay_0–3h_ or pfmdr1 copy number.

## Abbreviations

ACT: artemisinin combination therapy
ART: artemisinin
AS: artesunate
Asn: asparagine
ATV: atovaquone
CQ: chloroquine
DHA: dihydroartemisinin
HRM: high resolution melt
iRBC: infected red blood cell
LUM: lumefantrine
MFQ: mefloquine
NIF4: NLI interacting factor-like phosphatase
ORF: open reading frame
PCR: polymerase chain reaction
PPQ: piperaquine
QN: quinine
RBC: red blood cells
SNP: single nucleotide polymorphism
TRAC: Tracking Resistance to Artemisinin Collaboration
WGS: whole genome sequencing
RSA_0–3h_: ring-stage survival assay
PIP5K: phosphatidylinositol-4-phosphate 5-kinase
CNV: copy number variation
