# Supplementary material for: Artemisinin resistance without pfkelch13 mutations in Plasmodium falciparum isolates from Cambodia
Source: Malar J. 2017 May 12;16:195. doi: 10.1186/s12936-017-1845-5 (PMC5427620; doi:10.1186/s12936-017-1845-5)
Supplement: Supplementary file 1 — Additional file 1. Culture-adaptation of TRAC parasites. [file 12936_2017_1845_MOESM1_ESM.docx]

**Supplemental Methods**

**Culture-adaptation of TRAC parasites**
All parasite samples were collected under protocols approved by ethical review boards in Cambodia, at Oxford University and at the Harvard T. H. Chan School of Public Health. Culture-adaptation of parasites was accomplished by thawing cryopreserved material containing iRBCs that had been mixed with glycerolyte, with approximately 1.67 ml of glycerolyte added to every 1 ml packed RBCs. Samples were stored overnight in -80^o^C, and then transferred to liquid nitrogen for long term storage. Cryopreserved material volumes ranged from 0.5 to 5.0 ml. The entire tube was gently thawed by placement into 37^o^C water with gentle agitation by hand until the sample was just thawed. Immediately upon thawing the sample was transferred to a 50 ml conical tube and volume was measured. For every 1 ml of sample volume, a volume of 0.2 ml of sterile 12% NaCl (Fenwal, 4B7874Q) was added dropwise and the total sample gently mixed by swirling or partial rotation of the tube, and then incubated for 5 min at room temperature (RT) with gently mixing by partial rotation approximately two to three times during the incubation. For every 1 ml of original sample volume, a total of 9 ml of sterile 1.6% NaCl solution (Fenwal, 4B7870X) was added, the tube closed and inverted two to three times before incubation at RT for 2 min. Then, a total of 9 ml of 0.9% NaCl, 0.2% Dextrose (Fenwal, 4B7877X) was added to the tube for every 1 ml of original sample volume, and inverted once before centrifugation (2K, 5 min, RT). After aspirating the pellet, the sample was transferred to tissue culture dishes with fresh human blood (O+). For every 1 ml of original sample an individual tissue culture dish was established by adding 1 ml of 50% haematocrit O+ blood (freshly collected, and no more than seven days from collection) along with Hepes buffered RPMI media containing 12.5% AB+ human serum (heat inactivated and pooled). Cultures were placed in modular incubators and gassed with 1%O_2_/5% CO_2_/balance N_2_ gas (10 psi for 30-120 sec) and incubated with rotation (50 rpm) at 37^o^C. Cultures were settled for 30 min before changing media to retain the maximal amount of culture. Media was changed and smears made daily to monitor parasite growth. Of 67 cryopreserved samples attempted for culture adaptation, 66 were successful. One sample had evidence of bacterial contamination and was not utilized. The remaining 65 samples had visible parasites generally within one to two weeks’ time and were successfully established and utilized for this study. These cultures were returned to the TRAC project for community access, as per the collaboration agreement.
