# Supplementary figures and images for: Artemisinin resistance without pfkelch13 mutations in Plasmodium falciparum isolates from Cambodia
Source: Malar J. 2017 May 12;16:195. doi: 10.1186/s12936-017-1845-5 (PMC5427620; doi:10.1186/s12936-017-1845-5)

## Slide 1
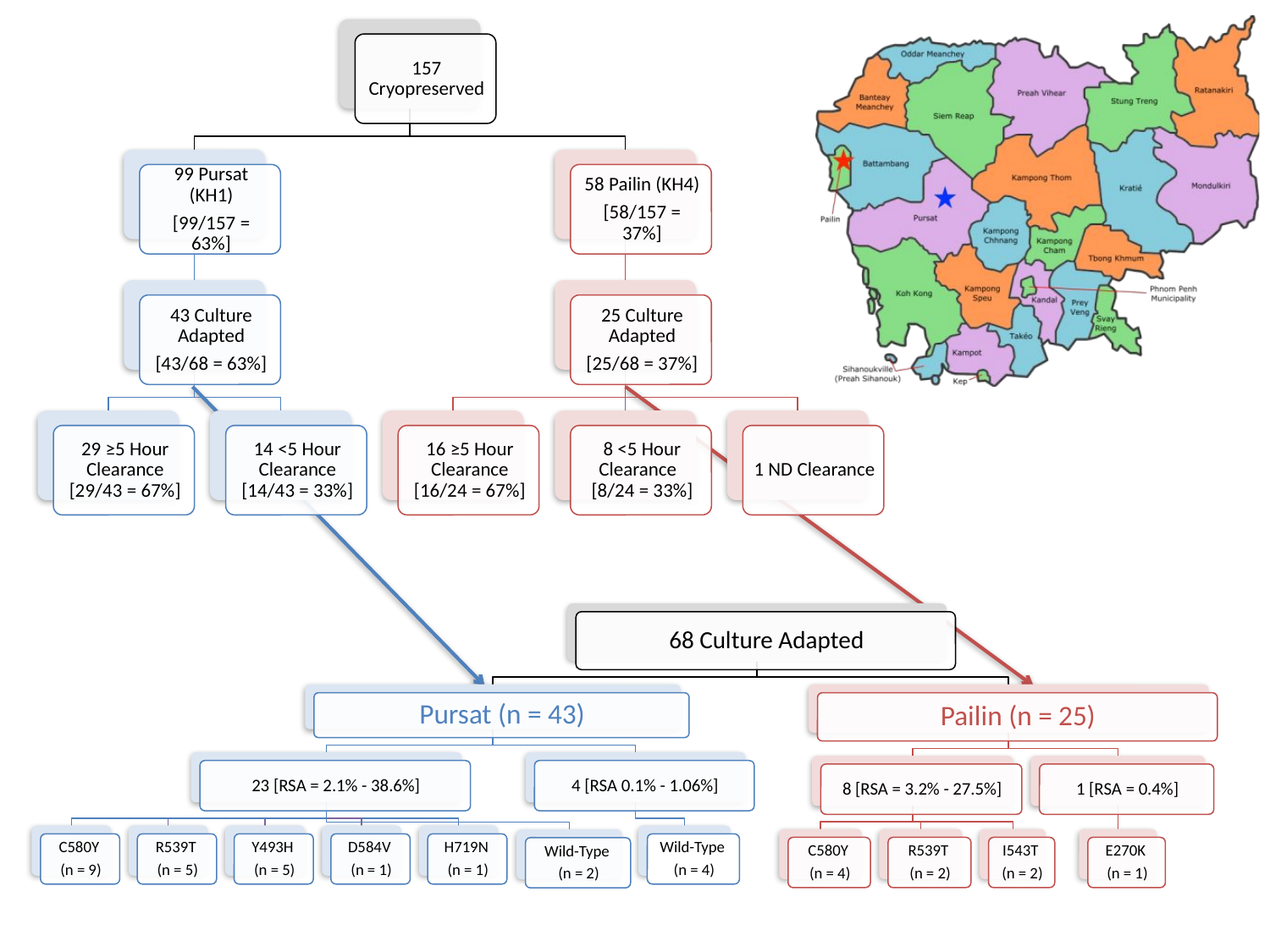

Supplement: Supplementary file 3 — Additional file 3. Schematic shows distribution of parasites obtained from Pursat (blue star on map, KH1 parasites indicated in blue) and Pailin (red star, KH4 parasites indicated in red) from Cambodia (map modified from https://commons.wikimedia.org/wiki/File:Cambodia_provinces_en.svg). [file 12936_2017_1845_MOESM3_ESM.pptx]
