# Supplementary material for: Artemisinin resistance without pfkelch13 mutations in Plasmodium falciparum isolates from Cambodia
Source: Malar J. 2017 May 12;16:195. doi: 10.1186/s12936-017-1845-5 (PMC5427620; doi:10.1186/s12936-017-1845-5)
Supplement: Supplementary file 4 — Additional file 4. Distribution of C580Y mutations in Cambodian isolates. Parasites from Pursat (round symbols) or Pailin (square symbols) were classified according to their Pfkelch13 alleles. Comparison of clearance half-life values (hours, h) from parasites harboring the wild-type (C) or mutant (Y) allele at amino acid position 580 in the Pfkelch13 locus, with culture-adapted parasites represented by black outlined symbols. C580 parasites harboring a mutation in the Pfkelch13 propeller domain other than C580Y are indicated by grey filled symbols. An unpaired t test was performed between the C580 and C580Y carrying parasites from Pursat (p < 0.0001) or Pailin (p < 0.05); with significant differences (p < 0.0001 overall) when both Pursat and Pailin were combined. [file 12936_2017_1845_MOESM4_ESM.pdf]

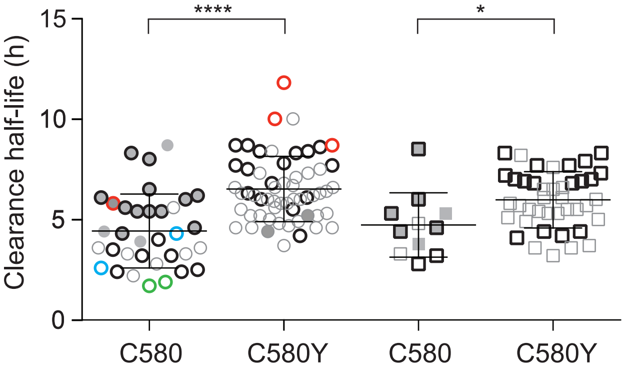

Additional file 3. Distribution of C580Y mutations in Cambodian isolates. Parasites from Pursat (round symbols) or Pailin (square symbols) were classified according to their *Pfkelch13* alleles. Comparison of clearance half-life values (hours, h) from parasites harboring the wild-type (C) or mutant (Y) allele at amino acid position 580 in the Pfkelch13 locus, with culture-adapted parasites represented by black outlined symbols. C580 parasites harboring a mutation in the Pfkelch13 propeller domain other than C580Y are indicated by grey filled symbols. An unpaired t test was performed between the C580 and C580Y carrying parasites from Pursat ($p < 0.0001$) or Pailin ($p < 0.05$); with significant differences ($p < 0.0001$ overall) when both Pursat and Pailin were combined.
