# Supplementary material for: Artemisinin resistance without pfkelch13 mutations in Plasmodium falciparum isolates from Cambodia
Source: Malar J. 2017 May 12;16:195. doi: 10.1186/s12936-017-1845-5 (PMC5427620; doi:10.1186/s12936-017-1845-5)
Supplement: Supplementary file 5 — Additional file 5. Association between percent survival RSA0–3h and clearance time. Correlation between clearance time on the x-axis and percent survival RSA0–3h on the y-axis. Each dot represents an isolate. Samples represented by color were used for in vitro drug testing and for pfmdr1 copy number variation determination—green for ART sensitive; blue for discordant; and red for ART resistant parasites. The grey dot marks the isolate with an E270K mutation. The dotted lines represent the cut off used to discriminate between resistant and sensitive parasites (1% for RSA0–3h, 4 or 5% for clearance). [file 12936_2017_1845_MOESM5_ESM.pdf]

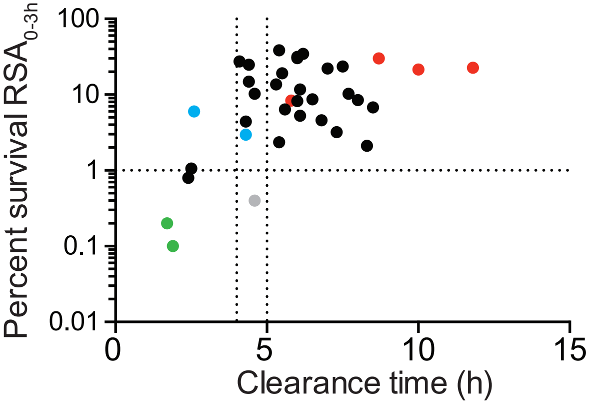

Additional file 4. Association between percent survival $RSA_{0\text{-}3h}$ and clearance time. Correlation between clearance time on the x-axis and percent survival $RSA_{0\text{-}3h}$ on the y-axis. Each dot represents an isolate. Samples represented by color were used for *in vitro* drug testing and for *pfmdr1* copy number variation determination—green for ART sensitive; blue for discordant; and red for ART resistant parasites. The grey dot marks the isolate with an E270K mutation. The dotted lines represent the cut off used to discriminate between resistant and sensitive parasites (1% for $RSA_{0\text{-}3h}$, 4 or 5% for clearance).
